# Long-Term Results after Treatment of Very Low-, Low-, and High-Risk Thyroid Cancers in a Combined Setting of Thyroidectomy and Radio Ablation Therapy in Euthyroidism

**DOI:** 10.1155/2013/769473

**Published:** 2013-07-09

**Authors:** Nikos Emmanouilidis, Harald Schrem, Michael Winkler, Jürgen Klempnauer, Georg F. W. Scheumann

**Affiliations:** Allgemein-, Viszeral- und Transplantationschirurgie, Hannover Medical School, Carl-Neuberg Straße 1, 30625 Hannover, Germany

## Abstract

*Introduction*. Differentiated thyroid cancer treatment usually consists of thyroidectomy and radio ablation in hypothyroidism 4-6 weeks after surgery. Replacing hypothyroidism by recombinant human thyroid stimulating hormone can facilitate radio ablation in euthyroidism within one week after surgery. The outcome of this approach was investigated. *Methods*. This is a prospective randomized trial to compare thyroidectomy and radio ablation within a few days after preconditioning with recombinant human thyroid stimulating hormone versus thyroidectomy and radio ablation separated by four weeks of L-T_4_ withdrawal. Tumors were graded into very low-, low- , or high-risk tumors. Recurrence-free survival was confirmed at follow-up controls by neck ultrasound and serum thyroglobulin. Suspected tumor recurrence was treated by additional radio ablation or surgery. Quality-of-life questionnaires with additional evaluation of job performance and sick-leave time were used in all patients. *Results*. Radio ablation in euthyroidism in quick succession after thyroidectomy did not lead to higher tumor recurrence rates of differentiated thyroid cancers in any risk category and was significantly advantageous with respect to quality-of-life (*P* < 0.001), sick-leave time (*P* < 0.001), and job performance (*P* = 0.002). *Conclusion*. Recombinant human thyroid stimulating hormone can be used safely and with good efficacy to allow radio ablation under sustained euthyroidism within one week after thyroidectomy.

## 1. Introduction

The standard treatment for differentiated thyroid cancer (DTC) consists of thyroidectomy followed by radio ablation therapy (RAT) [1–3]. For RAT an elevated thyrotropin (TSH) serum level of at least 30 mU/L is necessary to have a sufficient ^131^iodine uptake into remnant thyroid tissue. TSH levels of 30 mU /L and higher can be achieved either by complete thyroidectomy and four weeks of the L-T_4_ hormone withdrawal or by exogenous substitution of recombinant human TSH (rhTSH). If patients are preconditioned by L-T_4_ withdrawal, they have to cope with the increasing symptoms of hypothyroidism and the concurrent negative socioeconomic side effects [4–7]. The ability to substitute pituitary TSH by recombinant TSH uncouples the radio ablative treatment (RAT) from hypothyroidism and facilitates RAT in euthyroidism under full-scale L-T_4_ substitutive medication. The socioeconomic advantages of RAT in euthyroidism are a sustained quality-of-life and a significantly reduced number of sick-leave days [4, 8, 9] and it has been shown that rhTSH preconditioning is as effective and safe as preconditioning by hypothyroidism [10–13].

Here we present long-term follow-up data of a randomized clinical trial comparing the efficacy of RAT after preconditioning by rhTSH with hypothyroidism. In addition, we have measured and compared the impact of both competing strategies of preconditioning by rhTSH or hypothyroidism on quality-of-life and job performance. 

## 2. Subjects and Methods

### 2.1. Study Patients

Forty-four patients with a diagnosis of DTC (Table 1) gave written informed consent to participate in the study, which was approved by the institutional review board and ethics committee. Twenty-four patients were prospectively randomized for stimulation by rhTSH (female *n* = 18; male *n* = 6; mean age = 46.8 yrs.; SD ± 14.8 yrs.) and twenty patients for preconditioning by L-T_4_ abstinence (hypothyroidism) (female *n* = 15; male = 5; mean age = 55.6 yrs.; SD ± 13 yrs.). In the rhTSH group 15 (62%) patients were employees, 4 (17%) were housewives, and 5 (21%) were pensioners. In the hypothyroidism group 9 (45%) patients were employees, 6 (30%) were housewives, and 5 (25%) patients were pensioners. RhTSH patients received their first RAT on first hospitalization, while patients in the L-T_4_ withdrawal group were discharged from hospital and readmitted for the first RAT within 4–6 weeks after thyroidectomy while in a state of distinctive hypothyroidism. Tumors were staged following the UICC 2002 TNM classification and were categorized into very low, low-, and high-risk tumors following the UICC recommendations.

### 2.2. Questionnaire

All patients had to complete a questionnaire at five weeks after surgery. The questionnaire's main targets were clinical symptoms such as fatigue/lethargy, lack of concentration, disturbance of sleep/insomnia, intolerance to cold, cold skin, rough skin, slowed down movements, periorbital edema, and peripheral edema. Possible answers were *no*,* yes*, or *n.s.* (*not specified*) (Table 3). The patients were also asked whether they had an impaired job performance that was related to symptoms of hypothyroidism or not. Possible answers were* no*,* light*,* medium*,* strong*,* very strong, *or* not specified (e.g., pensioner)*. Housewives were asked to answer other than *not specified* (n.s.) (Table 4).

### 2.3. Recombinant Human Thyrotropin

RhTSH (Thyrogen, Genzyme, Cambridge, Mass.) with a biological potency of 10 U/mg of protein was used according to the manufacturer's instructions. Each vial containing 0.9 mg of rhTSH-alpha was dissolved in 1.2 mL of water for injection and administered by the i.m. route to the gluteal region 48 h and 24 h before RAT.

### 2.4. Radio Ablative Treatment (RAT)

After iodine uptake was confirmed by neck scan with 100 Milli-Becquerel (MBq) ^131^I, the ablative activity of 3700 MBq ^131^I was administered orally.

### 2.5. Laboratory Measurements

Serum levels of thyroxin (T_4_), 3,5,3′-triiodothyronine (T_3_), TSH, thyroglobulin (Tg), urinary iodine excretion, and urinary creatinine were measured on examination days. 

### 2.6. Scintigraphy

Whole body scans and scans of the neck region were conducted before RAT, at the time of RAT, and at three and twelve months after surgery in both groups with and without rhTSH. Additional scans were performed depending on the results of US examinations and Tg readings (Table 2). Scans at follow-up controls were performed after L-T_4_ withdrawal and using 100 to 600 MBq of ^131^iodine. Ablation was considered to be successful if less than 2.0% of the applied activity was taken up in the thyroid bed (TB) and no extrathyroidal uptake was noted. If a local DTC recurrence was suspected, for example, due to elevated Tg levels or due to ultrasound examination, a patient would receive an ablative activity of 3700 MBq ^131^iodine, even if the diagnostic scintigraphy beforehand was negative for tumor recurrence. Thus, diagnostic scans in these cases were performed using higher activities than the standard 100–600 MBq.

### 2.7. Ultrasound

Ultrasound (US) of the neck region was carried out at the time of RAT, at three- and twelve-month intervals after surgery, and subsequently at each follow-up examination. If the US revealed suspiciously enlarged lymph nodes or a suspicious mass paratracheal, an additional follow-up scintigraphy was planned and carried out shortly thereafter.

### 2.8. Statistical Analysis

Statistical analysis was performed using *IBM SPSS Statistics Ver.20* and *Analyse-it for Microsoft Excel* (*version 2.00*) (*Analyse-it Software*, *Ltd.* http://analyse-it.com/; 2007). Distribution for normality was tested on descriptive statistics. A nonparametric *U*-test (Mann-Whitney *U* test) was performed to test the significance of the underlying hypothesis. Survival data and tumor recurrence data were analyzed by *Kaplan-Meier* statistics and groups were compared using the *Log Rank* test.

## 3. Results

Patients were followed up for an average period of 52 months (median = 56 months; SE ± 2.8) (see also Table 2). In rhTSH receivers one patient had to be censored for the analysis of the quality-of-life queries due to a postoperative complication and prolonged postoperative weaning (see Table 3). With respect to the evaluation of therapy effectiveness three patients from rhTSH receivers and two patients of the hypothyroidism group had to be censored (see Table 2). 

In the group of rhTSH receivers, 13 (62%) patients were employees, 4 (19%) were housewives, and 4 (19%) pensioners. In the L-T_4_ abstinence group 9 (50%) patients were employees, 5 (28%) were housewives, and 4 (22%) patients were pensioners. 

The mean tumor size for rhTSH receivers was 20.4 mm (SD ± 14.4 mm), with one measurement for tumor size missing. The mean tumor size in patients preconditioned by L-T_4_ abstinence was 10.3 mm (SD ± 7.1 mm). Twenty-two rhTSH receivers had a histology of papillary differentiated carcinoma (PTC) and two had a follicular differentiation (FTC). In the hypothyroidism group eighteen patients had a PTC and two patients had a FTC. The rhTSH group and the hypothyroidism group had an equal distribution of high-risk, low-risk, very low-risk, and nonspecifiable-risk (X) tumors (Table 1).

**Figure 1:**
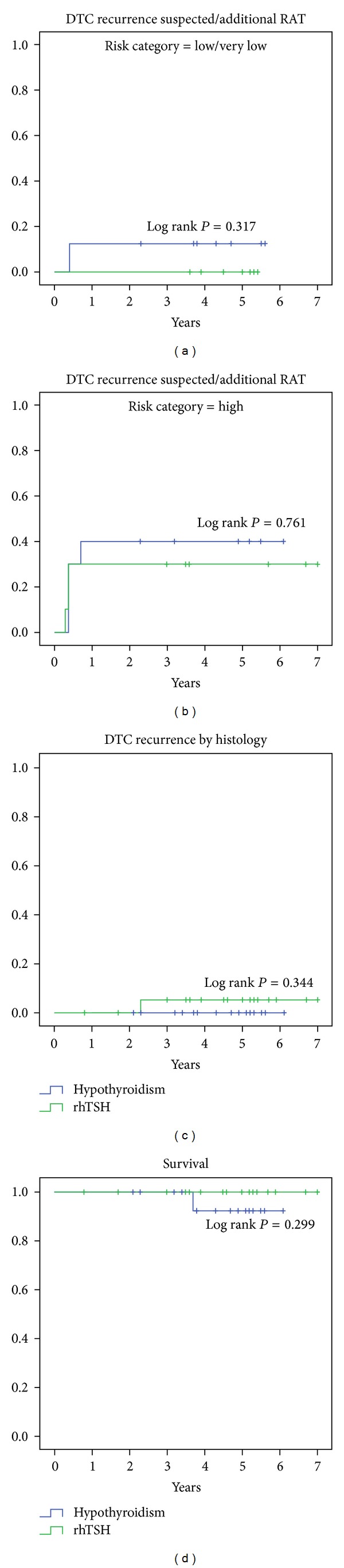
DTC-recurrence and patient survival.**  **Statistically there was no significant difference in numbers of suspected DTC recurrences in (a) low/very low and (b) high-risk DTC categories as well (*Log Rank P* = 0.317*; Log Rank P* = 0.761*, resp.*). (c)  Histological evidence (lymph node and bone metastasis) for DTC recurrence was found only in one case. (d) There was no difference in patient survival (*Log Rank P* = 0.299). One patient of the hypothyroidism group died due to natural cause. Prior to death this patient had neither a suspected nor histological proven DTC recurrence.

DTC recurrence was suspected in three rhTSH receivers and four patients after hypothyroidism (Figures 1(a) and 1(b)). Those patients with a suspected DTC recurrence were treated by an additional course of RAT. But only in one patient (rhTSH group) DTC recurrence proved to be true after surgery and histological examination of a supraclavicular lymph node (Figure 1(c)). This patient also received additional surgical procedures due to bone metastases. One patient died during follow-up without a suspected or a histological proven DTC (Figure 1(d)). Comparing preconditioning by rhTSH or hypothyroidism there was neither a statistically significant difference in numbers of suspected or histological proven DTC recurrences nor any significant difference in overall survival (Log Rank *P* > 0.05) (Figure 1).

The average time interval from surgery to primary ablation was 7.3 days for rhTSH receivers (SD ± 2.5; median = 7) and 31.4 days for patients preconditioned by hypothyroidism (SD ± 6.6; median = 31) (*P* < 0.0001). The average time interval from surgery to discharge from hospital after initial RAT was 10.1 days (SD ± 2.4; median = 9) for rhTSH receivers and 36.2 days (SD ± 4.7; median = 35) for hypothyroidism patients (*P* < 0.0001).

The average sick-leave time for rhTSH receivers was 4.8 days (SD ± 7.2; median = 0) and 40.7 days (SD ± 44.5; median = 28) for patients in the hypothyroidism group (*P* = 0.0350) (Table 1). Three patients in the rhTSH group reported sick-leave time after initial RAT and discharge from hospital: patient 3 reported 23 days of sick-leave time due to a necessary hospitalization not related to the DTC therapy; patient 11 reported 14 days of sick leave time, related to symptoms of hypocalcaemia; patient 6 reported 4 days of sick-leave time, related to a transient alteration of her voice and her job affiliation as a teacher. In contrast, all sick leaves of hypothyroidism patients were related to symptoms of hypothyroidism and no patient of this group reported any sick leave after the initial RAT. 

**Figure 2:**
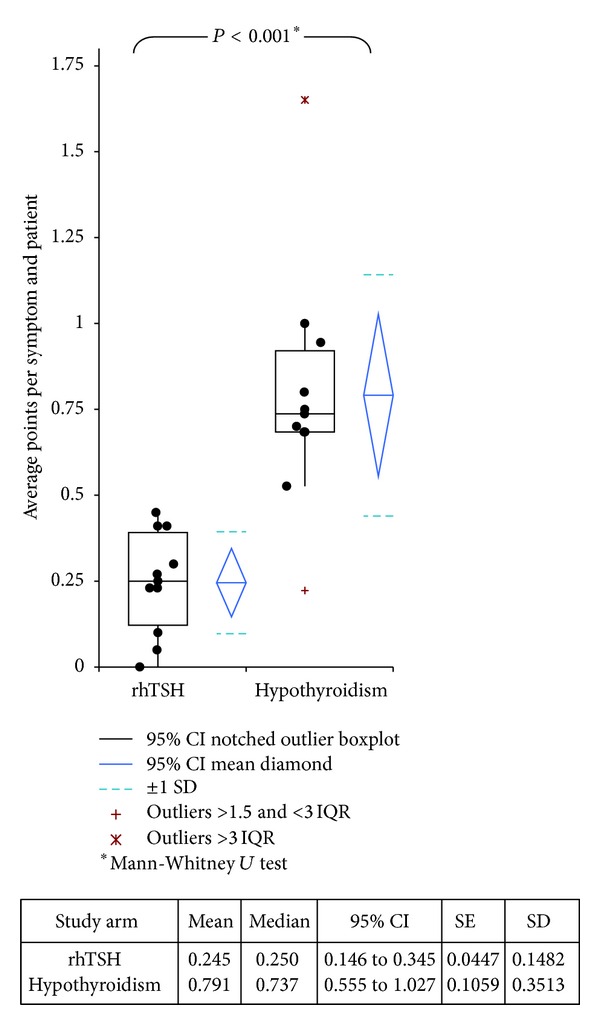
Symptoms of hypothyroidism.  Comparison of clinical symptoms for rhTSH receivers versus hypothyroidism patients by comparison of average points per category and patient. RhTSH patients had significantly fewer symptoms in comparison to standard protocol patients (*P* < 0.0001, Mann-Whitney* U *test).

Patients preconditioned by rhTSH experienced significantly fewer clinical symptoms in comparison to patients preconditioned by hypothyroidism (Table 3 and Figure 2). Almost all patients of the hypothyroidism group reported *fatigue or lethargy* (18/20; 90%), while only three patients of the rhTSH group (13%) experienced such symptoms. Two thirds (12/20; 60%) of the patients preconditioned by hypothyroidism experienced *lack of concentration* compared to only one patient of the rhTSH group (4%).

About one third of hypothyroidism patients experienced *intolerance to cold*, *gain in weight*, *disorder of sleep*, *constipation*, and *periorbital edema *which were reported by one third of the patients of the hypothyroidism group (6/20; 30% each). *Cold skin* was documented by four hypothyroidism patients (4/20; 20%). 

While *intolerance to cold* and *disorder of sleep* were each reported by four rhTSH patients (4/23; 17%), the symptoms *gain in weight* (1/23), *constipation* (1/23), *dry skin* (2/23), and *slowed down movements* (2/23) were not reported in significant numbers. *Cold skin* and *periorbital edema* were not mentioned in the rhTSH protocol group at all. 

In contrast to the patients of the rhTSH group, who reported that their treatment for thyroid cancer had no or only little impact on their job performance up to 5 weeks after surgery, nearly all patients of the hypothyroidism group—except three patients—reported a significant negative impact on their ability to maintain regular job performance (Table 4), which ultimately led to a significant increase of sick-leave days (Table 1).

## 4. Discussion

Today's standard in treatment of differentiated thyroid cancer (DTC) is a combination of thyroidectomy followed by radio-ablation therapy (RAT) [1–3]. To which extent the thyroid gland should be removed—total thyroidectomy *versus* a more limited thyroid resection and whether a central lymphadenectomy should be performed or not—is an on-going controversy [14, 15]. The benefit of RAT for a DTC recurrence-free survival though is undisputed. Before recombinant human TSH was available, the only way to achieve elevated thyrotropin levels for RAT was by thyroid-hormone abstinence after thyroidectomy. But hypothyroidism also meant that patients had to endure the debility and fatigue of a slowly developing hypothyroidism during this period [4–7]. Contrary, if a patient is preconditioned by rhTSH then L-T_4_-medication can be continued and RAT can be conducted in euthyroidism [6, 8, 11, 12, 16, 17]. Maintaining euthyroidism is not only beneficial to avoid the negative clinical symptoms of hypothyroidism and to avoid additional disadvantageous socioeconomic side effects, but is also beneficial for a normal wound-healing process [18–20]. Hence, from a patient's point of view it is understandable that rhTSH is favoured over L-T_4_ withdrawal when it comes to preconditioning for RAT. Nonetheless, the final long-term oncological outcome of the respective therapeutic strategy (rhTSH or L-T_4_ withdrawal) is still decisive for the choice of a treatment strategy for thyroid cancer.

In our clinic it was a common procedure that patients with a diagnose of DTC would receive their first RAT after a postoperative L-T_4_-abstinence period. This strategy was maintained until we had tested a shortened treatment protocol that utilizes rhTSH and merges surgery and radio-ablation therapy into one hospitalization period. When preconditioned by rhTSH, the consecutive primary RAT can be initiated within one week after thyroidectomy. This procedure was then compared to the common procedure of preconditioning by 4–6 weeks of L-T_4_-abstinence. In the initial study [21] we found that the lion's share of possible reduction of sick-leave time lies within the period from surgery to RAT. We also found that this improvement was achievable without any cut-backs on safety or ablation efficacy. 

Now, seven years after study initiation we report our long-term follow-up data of all 44 patients with the diagnosis of DTC (including *n* = 23 patients with high-risk tumors) who were prospectively randomized for either preconditioning by rhTSH or hypothyroidism. We have not found any significant difference between both preconditioning strategies with respect to DTC recurrence or survival, neither for the low/very low nor for the high-risk tumor categories (Figure 1) (*Log Rank P* = 0.317; *Log Rank P* = 0.761*, resp.*). The overall incidence of suspected DTC recurrences was comparable for both treatment strategies. As expected, the incidence of suspected DTC recurrence was a little higher for high-risk tumors as compared to the low/very low risk category (Figures 1(a) and 1(b)). We also confirm the possible reduction in overall treatment time of first-line therapy (thyroidectomy and RAT) down to 10 days, which equaled a time reduction of about 75% when compared to the standard procedure of preconditioning by hypothyroidism. Furthermore, sick-leave time was reduced significantly, if patients were preconditioned by rhTSH (~5 days) in comparison to patients who were preconditioned by hypothyroidism (~41 days). Last but not least, we have found that quality-of-life can be sustained during DTC treatment, if patients are preconditioned by rhTSH. That the impairment of job performance was significantly less for the rhTSH receivers in comparison to patients preconditioned by hypothyroidism (*χ* ^2^ *P* = 0.0015) is conclusive and supports the previous mentioned data. Overall, the ability to achieve a shortened treatment and sick-leave time while conducting RAT in euthyroidism without any cut backs in safety and therapy effectiveness by using rhTSH in the treatment of DTC of any risk category is as advantageous as it is simple and elegant. The public health-care system and health-insurance funds might have little interest to increase their expenses for any new drug-treatment strategy, especially when there is a conventional treatment strategy that works. Nevertheless, we hope that our data might trigger additional clinical trials focussing on this subject. More data might convince health care insurance companies and the community of doctors that this treatment strategy is not only beneficial for the patient, but in terms of “reduced sick-leave time” and a “sustained-productivity” is also beneficial for the health-care system and society in general.

## 5. Conclusion

We conclude that RAT after rhTSH preconditioning and in quick succession after thyroidectomy should be the standard procedure in the initial treatment of DTC—regardless of the DTC risk category.

## Figures and Tables

**Table 1:** 

Clinical data of all patients	rhTSH	Hypothyroidism	P*
Gender (M/F)	6/18	5/15	n.s.
Age [yrs.] (mean, median, range)	47, 50, 17–66	56, 58, 30–73	n.s.
Tumor histology (PTC/FTC)	22/2	19/1	n.s.
Tumor size [mm] (mean, median, range)	20.4, 20.0, 1–60	10.3, 8.5, 1.5–30	<0.01
pT			
1a	5	10	n.s.
1b	8	7	
2	5	1	
3	6	2	
pN			
X	4	0	n.s.
0	10	11	
1	10	9	
pM			
X	23	20	n.s.
1	1	0	
UICC 2002 staging			
X	4	0	n.s.
I	13	13	
II	0	0	
III	5	5	
IVA	1	2	
IVB	0	0	
IVC	1	0	
Risk category			
High	12	11	n.s.
Low	4	4	
Very low	5	5	
Sick leave* from surgery to first RAT (mean, median, range)			
(Days)	4, 0, 0–23	41, 28, 4–150	<0.001

*Mann-Whitney *U* test or Pearson *χ* ^2^ statistics applied where appropriate.

**Table 2:** Follow-up data of all patients.

Pat.	#	UICC 2002	Risk	Follow-up I			Follow-up II			Follow-up III			Follow-up IV			Follow-up V			Follow-up VI			Follow-up VI		
				Month	Reccur.	Therapy	Month	Reccur.	Therapy	Month	Reccur.	Therapy	Month	Reccur.	Therapy	Month	Reccur.	Therapy	Month	Reccur.	Therapy	Month	Reccur.	Therapy
rhTSH	11	I	X	4	No		12	No		19	No		32	No		44	No		55	No				
	23	X	X	3	No		10	No		f														
	24	X	X	3	No		5	No		13	No		20	No		f								
	3	I	Very low	3	No		12	No		p			p			p			p			64	No	
	4	I	Very low	4	No		13	No		p			p			p			p			63	No	
	13	I	Very low	3	No		13	No		20	No		29	No		37	No		47	No		61	No	
	15	I	Very low	2	No		13	No		24	No		38	No		f								
	18	I	Very low	4	No		12	No		18	No		30	No		P			54	No		f		
	5	I	Low	4	No		12	No		p			p			p			p			66	No	
	8	I	Low	3	No		p			p			p			p			p			64	No	
	14	X	Low	4	No		13	No		p			p			p			44	No		f		
	17	I	Low	SAE	No																			
	1	I	High	4	No		12	No		26	No		38	No		50	No		59	No		85	No	
	2	III	High	3	No		13	No		18	No		32	No		38	No		44	No		82	No	
	6	III	High	4	No		12	No		28	No		37	No		f								
	7	III	High	3	sus	RAT	10	No		28	LN	Surg	36	TB	Surg	36	oss	Surg	56	oss	Surg	59	oss	
	9	I	High	4	No		12	No		19	No		36	No		39	No		50	No		69	No	
	10	IVA	High	4	sus	RAT	8	No		17	No		25	No		38	No		57	No		72	No	
	12	I	High	4	sus	RAT	9	No		20	No		33	No		40	No		52	No		61	No	
	16	I	High	4	No		11	No		p			p			p			43	No		f		
	19	X	High	AE	No																			
	20	IVC	High	AE	No																			
	21	III	High	3	No		12	No		16	No		24	No		30	No		42	No		f		
	22	III	High	3	No		11	No		24	No		f											

Hypothyroidism	1	I	Very low	4	No		7	No		11	No		20	No		29	No		41	No		f		
	3	I	Very low	4	No		12	No		p			p			p			53	No		f		
	9	I	Very low	3	No		10	No		21	No		28	No		36	No		40	No		66	No	
	10	I	Very low	4	sus	RAT	9	No		21	No		24	No		f								
	15	I	Very low	AE																				
	4	I	Low	3	No		14	No		27	No		df											
	12	I	Low	4	No		12	No		30	No		f						p			67	No	
	13	I	Low	3	No		12	No		19	No		32	No		45	No		56	No		f		
	17	I	Low	3	No		12	No		18	No		f			45	No		f					
	2	I	High	3	No		13	No		25	No		32	No		44	No		57	No		f		
	5	III	High	4	sus	RAT	8	No		16	No		40	No		f								
	6	IVA	High	4	No		8	No		13	No		21	No		46	No		59	No		62	No	
	7	IVA	High	4	sus	RAT	12	sus	RAT	18	No		25	No		32	No		p			63	No	
	8	I	High	4	No		9	No		28	No		37	No		43	No		53	No		66	No	
	11	III	High	4	sus	RAT	8	No		24	No		30	No		36	No		50	No		62	No	
	14	I	High	4	sus	RAT	9	No		18	No		24	No		37	No		43	No		63	No	
	16	III	High	df																				
	18	I	High	3	No		12	No		20	No		27	No		f								
	19	III	High	4	No		12	No		16	No		23	No		37	No		43	No		55	No	
	20	III	High	4	No		12	No		18	No		32	No		38	No		f					

RAT: additional radio ablation therapy, Surg: surgery, sus: suspicion for tumor recurrence, LN: lymph node, oss: bone, TB: thyroid bed, AE: adverse event (e.g., protocol violation), SAE: serious adverse event (surgical complication), df: discontinued follow-up, f: follow-up in progress, p: patient did not show up for follow-up, Recurr.: tumor recurrence.

**Table 3:** 

Clinical symptoms	rhTSH		Hypothyroidism	
	Points per symptom	Avrg. points per symptom and patient	Points per symptom	Avrg. points per symptom and patient
Gain in weight	6	0.27	14	0.74
Fatigue/lethargy	9	0.41	33	1.65
Laps of concentration	5	0.23	20	1.00
Disorder of sleep/insomnia	10	0.45	14	0.70
Intolerance to cold	9	0.41	16	0.80
Constipation	5	0.23	15	0.75
Cold skin	1	0.05	10	0.53
Rough skin	5	0.25	17	0.94
Slowed movements	6	0.30	13	0.68
Periorbital edema	2	0.10	13	0.68
Peripheral edema	0	0.00	4	0.22

Avrg. points per symptom, patient, and study arm		**2.42**		**8.45**

Mann-Whitney's statistic	113.0			
*Z* statistic	—			
2-tailed *P*	**0.0002 (exact tables used, 27% ties)**			

**Table 4:** 

Impairment of job performance	rhTSH	Hypothyroidism
No questionnaire	1	0
Pensioner	5	5

No	**8**	**2**
Light	**6**	0
Medium	**4**	**5**
Strong	0	**6**
Very strong	0	**2**

Pearson's *χ* ^2^ statistic	17.58	
DF	4	
*P*	***0.0015***	
